# VIP152 is a selective CDK9 inhibitor with pre-clinical in vitro and in vivo efficacy in chronic lymphocytic leukemia

**DOI:** 10.1038/s41375-022-01758-z

**Published:** 2022-11-14

**Authors:** Steven Sher, Ethan Whipp, Janek Walker, Pu Zhang, Larry Beaver, Katie Williams, Shelley Orwick, Janani Ravikrishnan, Brandi Walker, Elizabeth Perry, Charles Gregory, Matthew Purcell, Alexander Pan, Pearlly Yan, Lapo Alinari, Amy J. Johnson, Melanie M. Frigault, Joy M. Greer, Ahmed Hamdy, Raquel Izumi, Xiaokui Mo, Deepa Sampath, Jennifer Woyach, James Blachly, John C. Byrd, Rosa Lapalombella

**Affiliations:** 1 Division of Hematology, Department of Internal Medicine, The Ohio State University College of Medicine, Columbus, OH USA; 2 Vincerx Pharma, Palo Alto, CA USA; 3 Department of Hematopoietic Biology & Malignancy, The University of Texas MD Anderson Cancer Center, Houston, TX USA; 4 Department of Internal Medicine, University of Cincinnati College of Medicine, Cincinnati, OH USA

**Keywords:** Cancer therapy, Leukaemia

## Abstract

Chronic lymphocytic leukemia (CLL) is effectively treated with targeted therapies including Bruton tyrosine kinase inhibitors and BCL2 antagonists. When these become ineffective, treatment options are limited. Positive transcription elongation factor complex (P-TEFb), a heterodimeric protein complex composed of cyclin dependent kinase 9 (CDK9) and cyclin T1, functions to regulate short half-life transcripts by phosphorylation of RNA Polymerase II (POLII). These transcripts are frequently dysregulated in hematologic malignancies; however, therapies targeting inhibition of P-TEFb have not yet achieved approval for cancer treatment. VIP152 kinome profiling revealed CDK9 as the main enzyme inhibited at 100 nM, with over a 10-fold increase in potency compared with other inhibitors currently in development for this target. VIP152 induced cell death in CLL cell lines and primary patient samples. Transcriptome analysis revealed inhibition of RNA degradation through the AU-Rich Element (ARE) dysregulation. Mechanistically, VIP152 inhibits the assembly of P-TEFb onto the transcription machinery and disturbs binding partners. Finally, immune competent mice engrafted with CLL-like cells of Eµ-MTCP1 over-expressing mice and treated with VIP152 demonstrated reduced disease burden and improvement in overall survival compared to vehicle-treated mice. These data suggest that VIP152 is a highly selective inhibitor of CDK9 that represents an attractive new therapy for CLL.

## Introduction

Chronic lymphocytic leukemia (CLL) is a genetically heterogeneous disease characterized by clonal expansion of B-lymphocytes that induce secondary immune suppression, cytopenias and organomegaly [1, 2]. Clinical outcomes of symptomatic patients meeting the iWCLL 2018 criteria for treatment have improved dramatically since the advent of small molecule inhibitors of B-cell receptor signaling, namely those of Bruton Tyrosine Kinase (BTK) [3–5]. Similarly, treatment with the B-cell lymphoma 2 (BCL-2) inhibitor venetoclax has shown improved progression-free survival, though not yet overall survival, as part of initial therapy. While most patients respond to targeted therapies directed at BTK or BCL-2 with little prejudice in response to sequence of administration, 15.5% of treatment naïve patients have been reported to develop refractory disease, justifying the need for novel therapies for refractory CLL [6–8].

Cyclin-dependent kinases (CDK), are a family of serine/threonine kinases which perform various roles in cellular function in tandem with their cyclin partner(s). Classically, these proteins were believed to regulate cell cycle progression; however, a subset of this class regulates transcriptional activity through signaling cascades [9]. CDK9, in complex with cyclin T1, forms the positive transcription elongation factor complex (P-TEFb). P-TEFb phosphorylates the second serine residue (S2) of the heptad repeat within the C-terminal domain of RNA Polymerase II (POLII) to initiate proximal-promoter pause releasing and the subsequent transcription of gene targets [9–13]. Of note, P-TEFb phosphorylation of POLII rapidly and dynamically increases the abundance of short-lived gene products (e.g. MYC, MCL1, PCNA, and cytokines), many of which are responsible for cell proliferation and survival [9, 14, 15]. MCL1 regulation is of particular importance in hematologic malignancies where leukemia is maintained through negative regulation of apoptosis [16, 17]. Indeed, multiple groups have demonstrated that a portion CLL patients with decreased sensitivity to venetoclax arise from a cellular dependence on MCL1, which can be overcome through inhibition of its expression [18, 19]. This clinical correlate has led to a vast area of research into the development of novel CDK9 inhibitors; however, many have not reached clinical application due to their narrow therapeutic index, toxicity profile, and overlapping inhibition of other CDK’s [20]. Of the many CDK9 inhibitors in development, we compared VIP152 activity to dinaciclib, KB-0742, and atuveciclib. Dinaciclib (SCH 727965), is a pan-CDK inhibitor whose function has been examined in solid tumor and hematologic malignancies [20–22]. KB-0742 is a CDK9 inhibitor with a narrower inhibitory profile and pre-clinical efficacy in prostate cancer and acute myeloid leukemia (AML) [23]. Atuveciclib (BAY1143572) is a selective, but less potent oral CDK9 inhibitor, which served as the forerunner for VIP152 [24].

The existing literature demonstrates both pre-clinical and clinical utility of CDK inhibitors in CLL [20]. Work done by our groups and others have shown the utility of flavopiridol, a non-selective CDK inhibitor, at inducing cell death and promoting partial clinical response in the setting of adverse genetic profiles [14, 25]. Flavopiridol monotherapy was active against CLL, but was limited by toxicity from off target kinases and the risk of hyperacute tumor lysis syndrome. Similarly, dinaciclib, a more potent and selective CDK inhibitor, demonstrated improved clinical activity with 64% of patients having a partial response with improved tolerability, though acute tumor lysis was still a risk [26]. Furthermore, Chen et al. recently explored the use of fadraciclib, a CDK2/9 inhibitor, in combination with venetoclax in primary chronic lymphocytic leukemia showing continued interest in the development of these inhibitors [27].

VIP152 is a potent and selective inhibitor of P-TEFb, whose synthesis and structure have recently been elucidated [24]. This compound, with its tolerable side-effect profile, has already shown preliminary clinical success in the context of double hit diffuse large B-cell lymphoma [28]. The clinical data in support of this compound drove us to investigate the biologic implications of CDK9 inhibition in CLL. In taking a multi-omic approach, we have demonstrated that CDK9 inhibition with VIP152 disrupts the highly ordered assembly of transcriptional machinery, thereby promoting cellular stress and inevitable cell death. Moreover, the decrease in P-TEFb regulated transcripts decrease the proliferative and survival signals that are essential for CLL maintenance. Overall, these findings provide the preclinical basis for the application of VIP152 in the context of CLL as well as provide evidence of future combinatorial opportunities.

## Methods

### Cell culture

HG-3 (DSMZ, Germany), MEC-1 (DSMZ, Germany), and OSU-CLL (The Ohio State University) were cultured in RPMI 1640 (Gibco) supplemented with 10% fetal bovine serum (FBS, VWR) and 1% penicillin/streptomycin/L-glutamine (P/S/G; Gibco) at 37 °C and 5% CO_2_. Human CLL cells were isolated and cultured as previously described [29–31]. Blood was obtained from CLL patients under an institutional review board-approved protocol with informed consent according to the Declaration of Helsinki. Cell lines were validated via short tandem repeat analysis by The Ohio State University Genomic Services Core, routinely tested for mycoplasma contamination (Universal Mycoplasma Detection Kit, ATCC 30–1012 K), and were discarded after passage twenty. *See supplemental materials for additional details*.

### Animal studies

All animal studies were carried out under protocols approved by The Ohio State University Institutional Animal Care and Use Committee (IACUC). Previously, 1 × 10^6^ splenocytes from Eµ-MTCP1 mice, which recently were described to mimic human CLL, were resuspended in PBS and injected via tail vein into male C57BL/6 J from The Jackson Laboratory [32]. Mice were monitored for development of peripheral disease by flow cytometry, measuring the percentage of CD5^+^/CD19^+^ cells in peripheral blood. Complete in vivo methods may be found in the supplementary material.

### lcRNAseq

CLL cells isolated from patients were sorted for CD5^+^/CD19^+^ into lysis buffer. Libraries were prepared and sequenced as previously described [33, 34].

### Nuclear CoIP proteomics

HG-3 cell line was treated with 1 µM VIP152 or DMSO for 2 h; nuclei were isolated using the Nuclear Complex Co-IP Kit (Active Motif) according to the manufacturer’s protocol. Nuclear lysate was immunoprecipitated using a CDK9 antibody (ab239364 - Abcam) or isotype control (3900 S – Cell Signaling Technology) with Dynabeads™ Protein G Immunoprecipitation Kit (10007D – ThermoFisher Scientific). Beads were submitted for proteomics to the Ohio State University Proteomics Center at the Campus Chemical Instrument Center. Full proteomic methods are available in the supplementary methods section.

### Western blotting

CLL cell lines (HG-3, MEC-1, and OSU-CLL) were cultured with VIP152 or DMSO control and lysed using RIPA buffer (CellSignaling Technology – cat. 9806) supplemented with PMSF. Lysate concentration was quantified using Pierce™ BCA Protein Assay Kit (ThermoFiser Scientific – cat. 23225) and proteins were analyzed via immunoblot. A full list of antibodies may be found in the supplementary methods section. All images are representative of three independent experiments.

### Statistical analysis

Statistical analysis of RNA sequencing data was performed by Dr. Pearlly Yan and Alexander Pan. Differences in gene expression were measured using Student’s *t*-test with Benjamini-Hochberg correction for multiple comparisons. All other statistical analyses were performed by the OSU Center for Biostatistics with SAS 9.4 (SAS Inc.). Differences between 2 groups, observing cells treated with vehicle or VIP152, were examined via paired Student’s *t-*test. Cytotoxicity data were analyzed via a mixed-effects model while taking into account repeated measures for each subject; multiple comparisons were corrected for using Holm’s method. The difference in survival between groups was analyzed with the log-rank test. *P* < 0.05 was considered significant.

## Results

### VIP152 selectively inhibits CDK9 with improved potency over other CDKs inhibitors

**Fig. 1:**
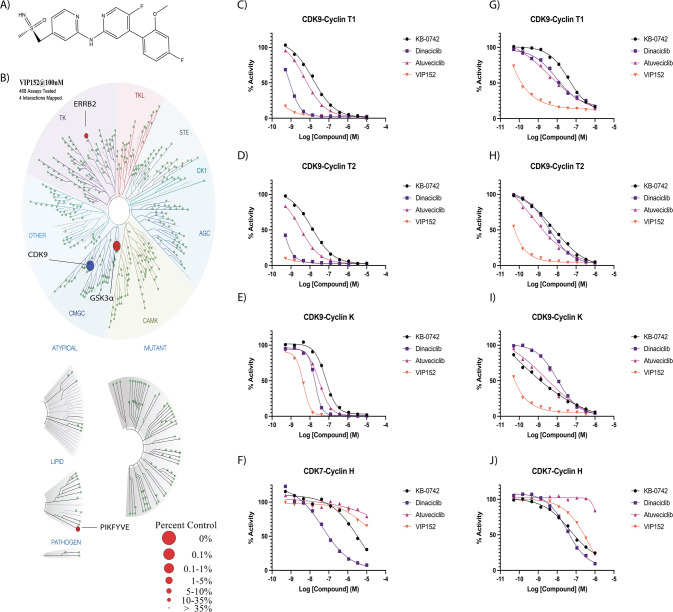
VIP152 is a selective CDK9 inhibitor with improved activity over other inhibitors. **A** Chemical structure of VIP152. **B** Inhibitory activity of VIP152 was plotted in a representation of the human kinome with results from the DiscoverX Kinome Scan, (**C**–**F**) Dose-response curves of VIP152, dinaciclib, KB-0742, and atuveciclib for CDK9 in complex with Cyclin T1/T2/K, CDK7 in complex with Cyclin H, in the radiometric acellular HotSpot Assay. **G**–**J** Dose-response curves of VIP152, dinaciclib, KB-0742, and atuveciclib for CDK9 in complex with Cyclin T1/T2/K, CDK7 in complex with Cyclin H, in the proximity-based cellular NanoBRET assay.

The structure and synthesis of VIP152 were recently described by Lücking et al. [24]. (Fig. 1A); however, the selectivity of the molecule in cellular systems has yet to be explored. We first sought to validate the inhibitory capacity of VIP152 at a global scale as seen by Lücking and colleagues [24]. When tested at 100 nM, VIP152 inhibited 95.3% of CDK9 activity compared with control (Fig. 1B). This represented inhibition of CDK9 7-fold greater than that of CDK7 at the same concentration (64% inhibition; Supplementary Table 1). At 100 nM, we also observed inhibition of GSK3α, PIKFYVE, and ERBB2 at 97.1%, 74%, and 65% activities relative to control, respectively. We also performed this experiment at 1000 nM, where CDK9 was inhibited at 99%. At 1000 nM, we also observed inhibition of IRAK1 (98.4%), CDK7 (97.9%), and ABL1 (96.8%). We found a strong concordance between our data and those of Lücking when comparing the most inhibited kinases. Potential off-target effects of VIP152 were next tested in vitro at a concentration of 10 µM for off-target activity against a broad range of receptors, transporters, ion channels, and enzymes (*n* = 138) using the Eurofins Diversity Panel. No significant (>50%) interference with any of these targets was detected except for rat imidazoline I2 (IC_50_ 1.4 µM), human Sigma σ1 (IC_50_ 1.78 µM), human adrenergic α2A receptor (IC_50_ 0.064 µM), α2B receptor (IC_50_ 1.18 µM), and α2C receptor (IC50 0.34 µM) (Supplementary Table 2).

We next compared VIP152 to other CDK inhibitors currently in development. Using the HotSpot assay, a radiometric assay with kinase specific substrates, we compared the inhibitory capacity of VIP152 in contrast to dinaciclib, KB-0742, and atuveciclib [21–23, 35]. The compounds were tested in a 10-dose series from 0.5 nM to 10 µM against CDK9 in complex with Cyclin T1/T2/K, CDK7/Cyclin H, and GSK3α/β. VIP152 showed higher potency for CDK9 when in complex with any of its cyclin binding partners, GSK3α, and GSK3β than any other inhibitor tested (Fig. 1C–E, Supplementary Fig. 1A, B). When measuring the inhibition of CDK7/Cyclin H, VIP152 and atuveciclib did not reach 50% inhibition of the complex, even at 10 µM; whereas KB-0742 and dinaciclib were characterized to have IC_50_’s of 2.37 µM and 87.2 nM respectively (Fig. 1F).

We additionally used the cell-based NanoBRET assay to characterize the selectivity of VIP152 in comparison to these same inhibitors (Fig. 1G–J, Supplementary Fig. 1C, D) [36]. While the NanoBRET revealed similar trends to the HotSpot assay in that VIP152 had higher potency for CDK9 complexes, we detected a greater degree of inhibition of CDK7/Cyclin H by VIP152 with this method; however, the IC_50_ was higher than dinaciclib and KB-0742. Taken together these data represent that VIP152 has a higher selectivity and potency for CDK9 and markedly lower CDK7 inhibition than other inhibitors.

### VIP152 decreases CLL proliferation and induces cell death via apoptosis

**Fig. 2:**
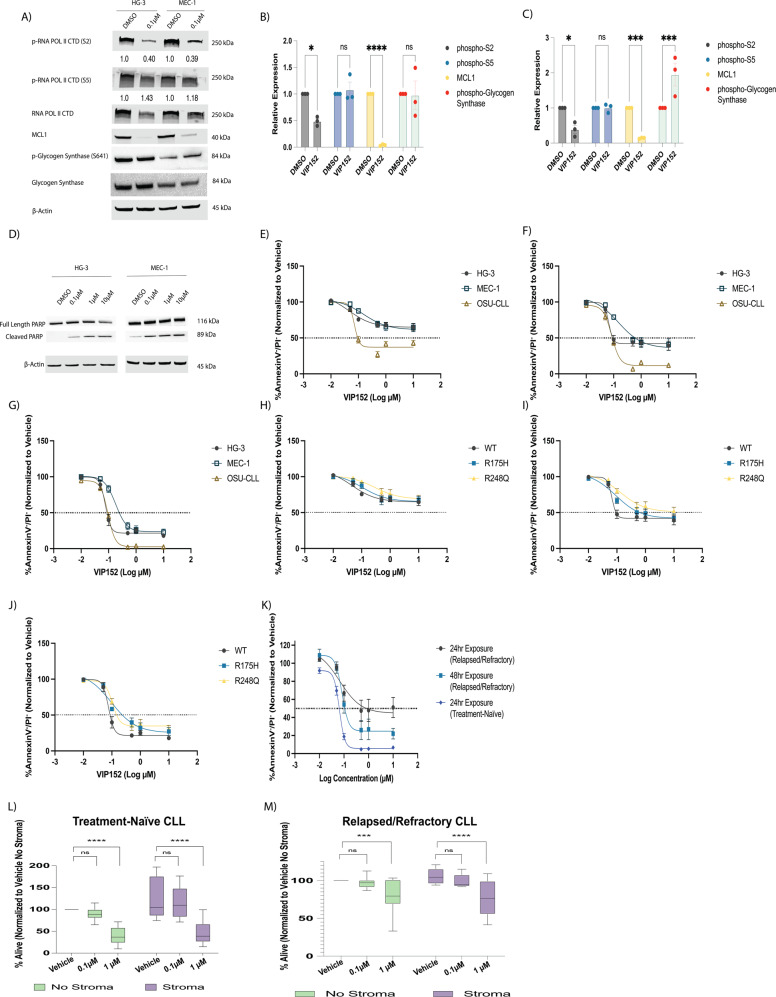
VIP152 inhibits proliferation and induces apoptosis in CLL cells. **A** Representative western blot of 8 h treatment of HG-3 and MEC-1 cell lines with DMSO or 0.1 µM VIP152. Densitometry analyses below bands indicate phosphorylation relative to total protein. **B**, **C** Densitometry analysis of three independent immunoblot experiments examining phospho-Serine 2 and phoshpo-Serine 5 residues of RNA Polymerase II, MCL1, and phospho-Glycogen Synthase upon VIP152 treatment in HG-3 (**B**) and MEC-1 (**C**). Results are shown as mean ± SEM. (*, ***, **** = *p* < 0.05, 0.001, and 0.0001 respectively). **D** Representative western blot of apoptosis induction in HG-3 and MEC-1 treated with VIP152 for six hours. Western blot is a representative image of three biological replicates. **E**–**G** Annexin V/propidium iodide flow cytometry dose-response curves of VIP152 treated HG-3, MEC-1, and OSU-CLL at 24 h (**E**), 48 h (**F**), and 72 h (**G**). Results are averaged of three independent biological replicates with results shown as mean ± SEM. **H**–**J** Annexin V**/**propidium iodide flow cytometry dose-response curves of VIP152 treated HG-3 TP53^WT^, HG-3 TP53^R175H^, and HG-3 TP53^R248Q^ at 24 h (**H**), 48 h (**I**), and 72 h (**J**). Results are averaged of three independent biological replicates with results shown as mean ± SEM. **K** Annexin V/propidium iodide flow cytometry dose-response curves of VIP152 treated treatment-naïve or relapsed/refractory patient samples for the listed timepoints. **L**, **M** Results of four-hour treatment with washout of 10 treatment-naïve (**L**) and 8 BTKi/venetoclax relapse/refractory (**M**) primary CLL patient samples plated with or without the human stromal cell line, HS5. (***, **** = *p* < 0.0005 and *p* < 0.0001 respectively).

To measure the protein effects of CDK9 inhibition with VIP152, we treated the HG-3 and MEC-1 cell lines with 0.1 µM VIP152 for 8 h (Fig. 2A–C). We observed a decrease in phospho-serine 2 (pS2) across both cell lines. Phosphorylation of serine 5, a marker of CDK7 activity was not diminished corroborating our previous experimental findings of the preferential inhibition of CDK9 complexes [37]. Furthermore, we did not observe a decrease in glycogen synthase phosphorylation at serine 641, a marker of GSK3α/β activity [38]. Consistent with CDK9 based inhibition of POLII, western blot analysis of MCL1 protein levels decreased with treatment. We also performed this experiment at a higher concentration (1 µM) across 24 h (Supplementary Fig. 2). Similarly, pS2, MCL1, and c-MYC decreased across the time course with minimal change to pS5. We additionally discovered that beginning at six hours, we were able to detect PARP cleavage by western blot (Fig. 2D) indicating induction of apoptosis [18, 30, 39].

To thoroughly investigate the anti-proliferative and cytotoxic effects of VIP152, we performed MTS analysis and annexin V/propidium iodide (AV/PI) flow cytometry (Fig. 2E–G and Supplementary Fig. 3, Supplementary Fig. 4A–F). HG-3, MEC-1, and OSU-CLL were cultured for 24, 48, and 72 h with VIP152 or DMSO. For our proliferation studies IC_50_s were determined to be 50–67 nM, 150–1000 nM, and approximately 100 nM for HG-3, MEC-1, and OSU-CLL respectively. A similar proliferation assay was performed with a two-hour drugging followed by washout and addition of the MTS reagent after 72 h. This experiment resulted in 50% growth inhibition for HG-3 (0.97 µM) and MEC-1 (1.7 µM; Supplementary Fig. 3) indicating that short pulse exposures can affect cell proliferation. Strikingly, while our MTS data suggest that HG-3 is the most susceptible to growth inhibition, Annexin/PI studies (Fig. 2E–G) show that OSU-CLL has the highest sensitivity to induction of apoptosis. Similarly to the proliferation studies, there was a significant dependence on time of exposure leading to apoptosis at lower doses of VIP152. Interestingly, beginning at 100 nM there appeared to be plateauing of apoptosis and proliferation where no additional increase in VIP152 concentration was associated with reduced viability or proliferation.

Given that deletions of 17p and mutations in *TP53* are associated with decreased survival, we wanted to look at the cell killing effects of VIP152 in these samples. To assess this, *TP53* R175H and R248Q mutated HG-3 cell lines were tested for their susceptibility to cytotoxic activity with VIP152 [40]. Clonal populations with R175H and R248Q mutations were treated for 24, 48, and 72 h with VIP152 and proliferation and apoptosis were measured by MTS and Annexin/PI respectively (Fig. 2H–J, and Supplementary Fig. 4D–F). Despite the presence of critical stabilizing mutations in R175H and R248Q in the p53 protein, VIP152 is effective at inducing significant levels of cytotoxicity in this difficult to treat subtype of CLL. We observed that with increasing time of exposure the mutant cell lines became more responsive to VIP152. Of note, our proliferation data do show that the plateau phenomenon seen in the wildtype HG-3, MEC-1, and OSU-CLL are not recapitulated in the P53 mutants. These data may suggest independent anti-proliferative and cytotoxic roles of CDK9.

We next tested the cytotoxic activity of VIP152 in 10 treatment-naïve primary CLL samples and 8 primary samples derived from patients who have relapsed or were refractory to BTK inhibitor therapy and venetoclax (Fig. 2K). The relapsed/refractory samples used in these experiments were taken from patients that were heavily pre-treated, with many bearing concomitant mutations (*BTK* and *TP53*), and often poor prognostic cytogenetic markers (del(11)(q22q23) and trisomy 12). Indeed, seven of the eight patient samples bore mutations in *TP53* with variant allele frequencies ranging from 6.4% to 91.1% (Table 1). We observed treatment-naïve samples as being more sensitive to VIP152 inhibition with an IC_50_ of <100 nM. The relapsed/refractory samples did demonstrate 50% killing at 24 h (IC_50_ ~1 µM), and this improved in 48 h treatment.

**Table 1:** Clinical information for relapsed/refractory CLL patient samples.

Patient No.	Sex	Age (*yr*)	Prior Therapies	Cytogenetic Features	Identified Mutations of Interest	*TP53 Mutant* VAF (%)
1	Male	49	10	46,XY,del(11)(q22q23)[cp2]/46,sl,t(14;22)(p12;q11.2)[6, three w/nonclonal abnormalities]/46,XY,der(11)t(11;11)(p15;q12)del(11)(q22q23),der(11)t(11;11)(p15;q12)[8]/46,sdl2,del(4)(q31),add(9)(q22),-der(11)t(11;11),+add(11)(q22)[1]/46,XY[3]	C481S mutation in BTK P2514fs mutation in NOTCH F1142L mutation in PLCG2 I195N mutation in TP53	19.5 (I195N)
2	Female	54	10	45,XX,der(1)t(1;8)(q32;q13),del(11)(q22q23),add(12)(p13),−13[3,one w/nonclonal abnormalities]/46,XX[17]	C481S & T474I mutations in BTK C730 mutation in ASXL1 L603fs mutation in BIRC3 E571K mutation in XPO1	6.4
3	Female	49	7	45,XX,der(1)t(1;8)(q32;q13),del(11)(q22q23),add(12)(p13),−13[3,one w/nonclonal abnormalities]/46,XX[17]	C481S mutation in BTK D832 mutation in ASXL1 V216M & R209fs mutations in TP53	78.4 (V216M) 22.1 (R209fs)
4	Female	43	5	42–45,XY,−4,−5,−11,psu dic(15;11)(q26;p11.2),add(17)(p11.2),add(19)(q13.4),+mar1,+mar2,+mar3[cp6]/44–46,sl,der(1)add(1)(p36.1)add(1)(q21),der(6)t(1;6)(q21;q21),−9,-psu dic(15;11),+15,+11,-add(19),+19,+r,-mar1,-mar3,+mar4,+mar5[cp5]/46,XX[7]/nonclonal[2]	D855fs mutation in ASXL1 T663I mutation in SF3B1 R415P mutation in ZRSR2 S215I mutation in TP53	91.1 (S215I)
5	Male	53	7	45,XY,der(3)t(3;13)(p25;q14.3),del(11)(q13q23),t(11;12)(q23;q15),add(13)(q12),dic(15;17)(q11.2;p11.2)[4, one is 4n]/45,idem,−8,+der(?)(22qter->22q11.2::?::8q21->8qter)[6,one w/nonclonal abnormalities]/44,idem,−8,add(10)(p13),+mar[1]/ 44,idem,−8,−13,+add(13)(p11.2),add(16)(q12)[2]/ 44,idem,add(2)(q37),add(5)(p13),del(6)(q21q25),−8,-del(11),+der(11)add(11)(p15)del(11)(q13q23)[10, 3 w/nonclonal abnormalities]/ 45,X,-Y[8]/ 46,XY[8]/nonclonal[1]	Q2444 mutation in NOTCH1 Splice Donor Site Mutation in TP53	90.0 (c.782 + 2 T > A)
6	Male	43	20	Stimulated: 46,XY,add(4)(p14),del(11)(q22q23)[14,one w/nonclonal abnormalities]/46,XY[5]/nonclonal[1] Unstimulated: 46,XY,add(4)(p14),del(11)(q22q23)[2] (2019)	C481S mutation in BTK E571A mutation in XPO1 V600E mutation in BRAF K700E mutation in SF3B1	No Mutation in TP53
7	Male	52	10	46,XY,del(11)(q13q23)[17,one w/nonclonal abnormalities]/45,sl,del(X)(q22q26),−1,der(3)t(1;3)(p13;p25),t(4;18)(p15;p11.2),dic(8;17)(p11.2;p11.2),add(11)(p15),del(13)(q12q14),+mar[3]	C481S mutation in BTK E1006 mutation in ASXL1 E433 mutation in BIRC3 G469A mutation in BRAF Y266S mutation in PTPRD I195T mutation in TP53	16.1 (I195T)
8	Female	51	5	43–53,XX, + X, + add(3)(p11.2),+4,+5,−6,del(6)(q21q27),add(7)(p13),add(7)(q36),dic(9;15)(p11.2;p11.2),+11,+add(12)(q13),−16,−17,add(19)(q13.3),del(21)(q22),add(22)(q13),+1–8mars[cp20] (2021). 47,XX, + 12[cp2]/47,sl,+1,dic(1;17)(p12;p11.2),add(2)(p13),add(5)(q13),add(9)(p13),del(9)(p22),add(11)(q23),add(12)(q24),add(13)(q34)[1]/47,sdl,add(14)(q32)[7, one is 4n w/ nonclonal]/46,XX[8]/nonclonal[2] (2020)	P2514fs mutation in NOTCH1 E76G mutation in PTPN11 L1155I mutation in ASXL1 D281G mutation in TP53	85.5 (D281G)

We next looked at the cytotoxic activity of VIP152 primary CLL samples with or without a human stromal cell line (HS-5) co-culture. The cytoprotective effect of the stromal microenvironment on CLL cells has been reported by multiple groups and represents a more physiologically relevant pre-clinical screening approach [41–43] to better predict in vivo activity. CLL cells were treated with VIP152 or DMSO for four hours, replacement media added, and AV/PI flow cytometry was performed after 24 h (Fig. 2L, M, Supplementary Fig. 5A). Treatment with 1 µM of VIP152 demonstrated statistically significant cell killing, which overcame stromal protection with a difference in viability of 82% between DMSO and VIP152 (*p* < 0.0001). Additionally, to investigate if the cytotoxic effect was due to impaired HS-5 function with VIP152, a separate analysis was performed with independent drugging of CLL samples and plating on undrugged HS-5 (Supplementary Fig. 5B). These data demonstrated similar findings to the full co-culture system wherein a 1 µM drugging induced significant cell death with a difference in viability between 1 µM and DMSO of 67.21% (*p* = 0.0248).

Consistent with our data from treatment-naïve samples and data from our *TP53* mutated cell lines, a four-hour exposure of VIP152 was able to induce significant apoptosis after 24 h and overcome stromal protection in the relapse/refractory setting (Fig. 2M). Irrespective of *TP53* mutational status or previous treatments, we observed that VIP152 inhibits proliferation and induces potent cell death, which can overcome stromal protection. Moreover, our data indicate that VIP152 may serve as a potential therapeutic for patients who have previous resistance to multiple targeted therapies for which there are few options available.

### VIP152 rapidly alters the transcriptome and disrupts cellular pathways

**Fig. 3:**
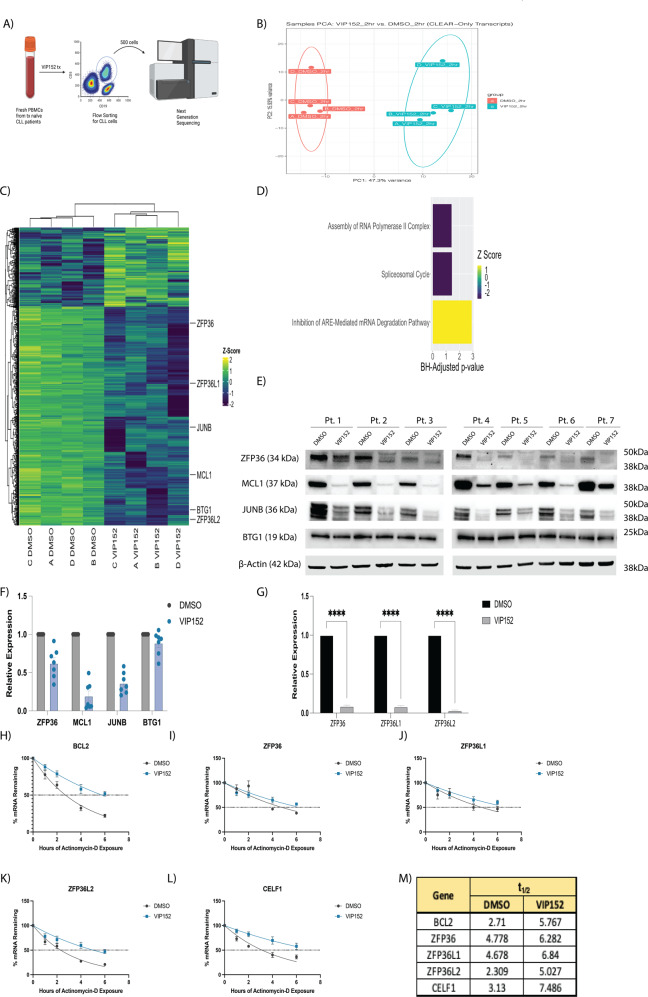
VIP152 induces rapid transcriptomic changes. **A** Schematic cartoon of limiting-cell RNA-seq experiment. **B** Principal component analysis of RNA sequencing data. Each dot represents a unique patient sample treated with either DMSO (red) or 1 µM VIP152 (teal). **C** Heat map of most differentially expressed genes. Genes selected by overall DEG’s with a LFC > 1 or LFC < −1 adj. *p*-value < 0.05. **D** Plot of pathways from Ingenuity Pathway Analysis with *p*-value < 0.05 and *z*-score > 0 or *z*-score < 0. **E**–**G** 7 CLL patient samples were treated for 8 h with 1 µM VIP152 and then analyzed for protein expression by western blot (**E**, **F**) and ZFP36, ZFP36L1, and ZFP36L2 expression by qRT-PCR (**G**). **H**–**L** 5 CLL patient samples were treated for 8 h with 1 µM VIP152 and then supplemented with 500 nM Actinomycin-D. RNA was taken at varying timepoints and analyzed via qRT-PCR**. M** mRNA half-lives of analyzed transcripts in (**H**–**L**) as determined by nonlinear regression using a one-phase decay method.

P-TEFb’s role in regulating transcription through POLII CTD phosphorylation is a canonical function of this complex [9, 10, 12, 44]. To interrogate the transcriptional changes associated with VIP152 treatment, we treated B-cells, isolated from four patients with treatment-naïve CLL, with 1 µM VIP152 or DMSO for two hours followed by fluorescence-assisted cell sorting for 500 live (Sytox^−^), CD45^+^/CD5^+^/CD19^+^ cells (Fig. 3A). Our group has previously defined an analysis pipeline, named CLEAR, which identifies evenly distributed reads for differentially expressed gene (DEG) analysis [34]. Principal component analysis showed separation by treatment group with minimal change associated with each patient sample (Fig. 3B). We observed an increase in downregulated transcripts (759 transcripts; log_2_ fold Change < −1 and BH-corrected *p*-value < 0.05) compared to upregulated transcripts (285 transcripts; log_2_ fold change >1 and BH-corrected *p*-value < 0.05; Supplementary Fig. 6; Supplementary Table 3). When we looked at the expression of these differentially abundant genes via an unsupervised clustered heatmap, we noted that there was high concordance between the patient samples with clustering dependent on treatment group (Fig. 3C). The treatment did induce decreased transcript abundance of genes previously reported to be controlled by CDK9 activity, namely JUNB, BTG1, and MCL1 [45, 46]. Downregulation of the anti-apoptotic MCL1 (LFC: −1.231056 & *p*-value = 0.02) likely contributes to the susceptibility of primary CLL cells to apoptosis (Fig. 2F, G) [18]. Indeed, western blot analysis of patient samples treated with VIP152 demonstrate a decrease in MCL1 and JUNB protein but not BTG1 (Fig. 3E, F). Gene set enrichment analysis identified translation machinery to be enriched in the treatment group relative to control; although this was not maintained functionally as demonstrated by no abundant change in S6 ribosomal protein phosphorylation of treated CLL primary samples (Supplementary Fig. 6B–E). Pathway analysis of the DEGs with Ingenuity Pathway Analysis revealed an upregulation in transcripts belonging to the “Inhibition of ARE-Mediated mRNA Degradation” pathway (Fig. 3D). It has been established that AU-rich element (ARE) mRNA molecules are under tight temporal regulation and control a variety of cellular processes [47, 48]. Moreover, the relationship between P-TEFb mediated transcription and ARE-bearing mRNA has previously been described [49]. To further probe this relationship, we performed western blot analysis on seven CLL patient samples and observed decreased expression of tristetrapolin (ZFP36), a key regulator of ARE-bearing mRNA degradation, with VIP152 treatment (Fig. 3E, F). qRT-PCR of these patient samples and the HG-3, MEC-1, and OSU-CLL cell lines supported this downregulation at the transcript level as well as that of ZFP36L1 and ZFP36L2 (Fig. 3G and Supplementary Fig. 6F). We appreciate from our limiting-cell RNA sequencing experiments that the loss of tristetrapolin (ZFP36) transcripts (LFC: −4.02; adjusted *p*-value: 7.97*10^−40^) and the upregulation of ribosomal transcripts, likely correspond to a possible compensatory mechanism by which these cells attempt to improve translational output and maximize transcript maintenance in response to VIP152-induced transcriptional shutdown [49]. While translational activity was not upregulated in our experiment, we turned to look at the transcripts regulated by ZFP36 and asked if its loss would lead to a dysregulation of those transcripts’ degradation. We looked at transcripts which had been shown by photoactivatable ribonucleoside cross-linking and immunoprecipitation (PAR-CLIP) to interact with ZFP36 [50]. The dataset revealed that ZFP36 protein interacts with its own transcript as well as the transcripts of other closely-related RNA regulators (e.g. ZFP36L1, ZFP36L2, and CELF1). Additionally, we chose to look at BCL2 transcript stability as ZFP36L1 has been shown in B cell lymphoma to negatively regulate BCL2 transcript stability [50]. We performed an mRNA stability study on patient samples treated with VIP152 with Actinomycin-D and observed an increase in the half-lives of each of these transcripts including BCL2 (Fig. 3H–M). Additionally, we performed this experiment looking at two transcripts which do not bear an AU-Rich element as a negative control and indeed did not see an increase in mRNA half-life (Supplementary Fig. 7). These data provide a functional relationship between CDK9 and the stability of AU-Rich element bearing transcripts sensitive to ZFP36 family member dysregulation.

### VIP152 based inhibition decreases P-TEFb protein–protein complex formation

**Fig. 4:**
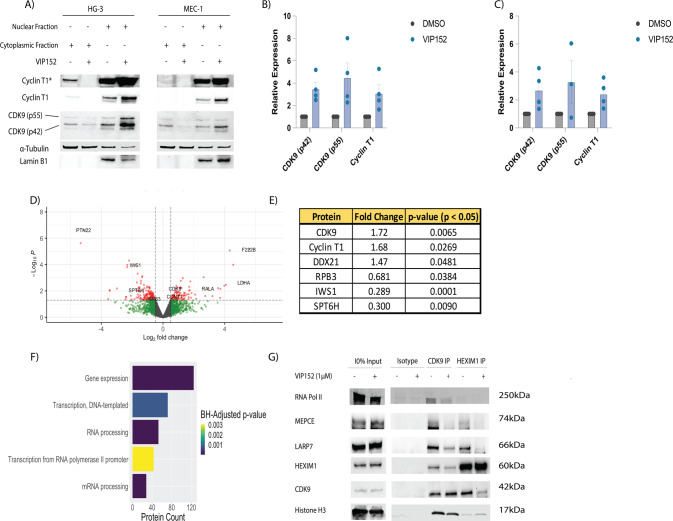
VIP152 disrupts P-TEFb canonical binding partners. **A**–**C** Cytoplasmic and nuclear extracts of HG-3 and MEC-1 treated with 1 µM VIP152 for two hours with quantification (**B**, **C**). **D**, **E** Proteomic characterization of nuclear immunoprecipitation of VIP152 treated HG-3 cells. Gray dots indicate non-differentially associated proteins. Green dots indicate transcripts with a LFC > 0.5 or LFC < −0.5 but *p*-value > 0.05. Blue dots indicate proteins with a *p*-value < 0.05 but −0.5 < LFC < 0.5. Red dots indicate proteins with a *p*-value < 0.05 and a LFC > 0.5 or LFC < −0.5. **D** Volcano plot of differentially associated proteins. **E** Table of P-TEFb and POLII-associated proteins. **F** Plot of pathways from Ingenuity Pathway Analysis with *p*-value < 0.05 and *z*-score > 0 or *z*-score < 0 (**G**) Nuclear IP of CDK9 and HEXIM1 of VIP152 treated HG-3 cells probing for components of the 7SK-RNA complex.

Having observed the transcriptomic effects of VIP152 inhibition, we hypothesized a change in the P-TEFb complex would occur validating the mechanism of this agent. Previously, Dow et al. described changes in P-TEFb nuclear complexes with flavopiridol treatment; specifically, they observed loss of nuclear speckles of P-TEFb complexes containing HEXIM1, a major component of the 7SK-RNA complex that inhibits P-TEFb [51]. We observed an increase in the 42 kDa and 55 kDa isoforms of CDK9, as well as Cyclin T1, in the nuclear fraction following treatment (Fig. 4A, B). Data from RNA sequencing (Fig. 3) and cell line drugging experiments (Supplementary Fig. 8) did not demonstrate changes to CDK9/Cyclin T1 expression at a global level. Next, a nuclear immunoprecipitation of CDK9 after a two-hour drugging in the HG-3 cell line (*n* = 3) was performed, followed by liquid chromatography with tandem mass spectrometry (LC-MS/MS). We detected 226 differentially co-localized proteins and again observed an enrichment in the treated fraction for P-TEFb (Fig. 4B, C). Additionally, we observed a decreased association with transcription-associated machinery (SPT6H and IWS1) and a component of the core complex of POLII (RPB3). The decreased association with SPT6H and IWS1 are to be expected given the dependence of these proteins on S2 phosphorylation for their association [52–57]. These findings were validated via western blot in the MEC-1 and HG-3 cell lines (Supplementary Fig. 9). Pathway analysis of the significantly differentially associated proteins revealed that the targets belong to the categories of gene expression, transcription, and RNA processing (Fig. 4D). Finally, to appreciate the effect of inhibition on the association with the inhibitory 7SK-RNA complex, we performed reciprocal nuclear co-immunoprecipitation of CDK9 and HEXIM1 (Fig. 4E and Supplementary Fig. 10). We found that treatment resulted in decreased association of CDK9 with components of the complex (HEXIM1, MEPCE, LARP7) in HG-3 and MEC-1. This decrease in 7SK association due to inhibition is consistent with other work characterizing the disruption of the 7SK complex as a result of cellular stresses [58, 59]. Indeed we observe from our proteomics an increase in association with DDX21 (Fold Change = 1.47, *p*-value < 0.05) which has been shown to reorder the 7SK-RNP to release P-TEFb for local association with POLII [59]. Taken together, these data suggest that VIP152 disrupts P-TEFb association with its binding partners, and combined with the decreased association with POLII disrupts the kinase cascade necessary for POLII activity.

### VIP152 improves survival and decreases disease burden in a murine CLL model

**Fig. 5:**
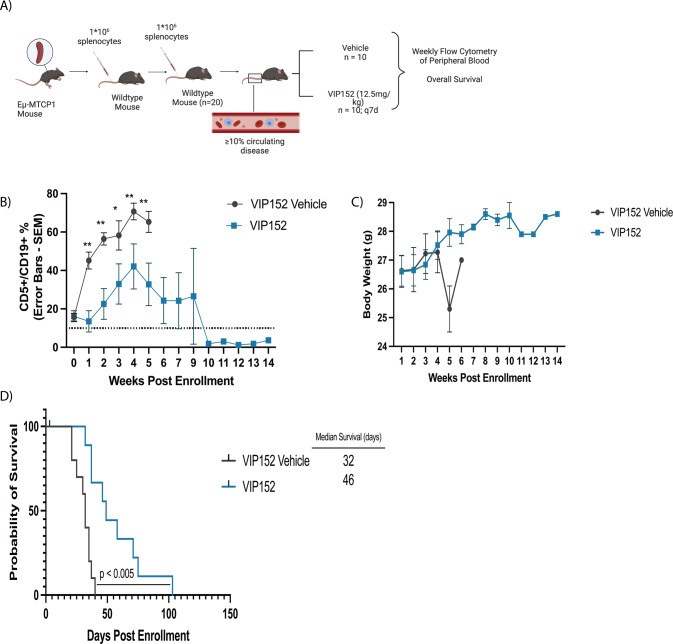
VIP152 improves survival in the Eµ-MTCP1 CLL model. 20 C57BL/6 J mice were engrafted via tail vein with 10^6^ splenocytes from leukemic Eµ-MTCP1 mice. **A** Schematic cartoon of in vivo adoptive transfer study. **B**–**D** Measurement of peripheral disease as characterized by CD45^+^/CD5^+^/CD19^+^ % in peripheral blood (**B**), weight monitoring (**C**), and survival (**D**) of mice following enrollment upon either VIP152 (blue) or vehicle treatment (red). 10 mice were enrolled into each treatment arm. (*,** = *p* < 0.01 and *p* < 0.005 respectively). Error bars indicate mean of surviving mice ± SEM.

In vitro systems generally lack the full microenvironment that can lead to tumor cell resistance and survival. Next, we sought to assess the in vivo efficacy of VIP152 to better evaluate its potential clinical utility in patients with CLL. To accomplish this we used a novel transgenic model of CLL (Eµ-MTCP1 model) developed recently by our group that is characterized by CD5^+^/CD19^+^ and B220^Dim^ expansion in the blood [32]. Splenocytes from diseased Eµ-MTCP1 mice were serially engrafted via tail vein injection and monitored for leukemia via weekly flow cytometry (Fig. 5A). Mice were randomized to vehicle versus VIP152 at the time blood disease was evident and treated every week until endpoint. Notably, mice enrolled into VIP152 treatment maintained a statistically significantly lower CD5^+^/CD19^+^ disease burden throughout the duration of the study (Fig. 5B). We did not observe any weight loss over the course of study for the VIP152-treated mice (Fig. 5C), consistent with previously published work [24]. Mice in the VIP152 treatment group had improved overall survival compared with vehicle with a median survival of 46 days vs 32 days respectively (Fig. 5D – *p* < 0.005). Following enrollment two mice had reduction in disease that was sustained below 10% for the duration of study and these mice were censored after 100 days upon which treatment was ceased. Two months following treatment cessation these mice succumbed to likely illness with evident splenomegaly. Additionally, mice in the treatment group did not have significantly smaller spleens nor significant decrease in spleen disease burden at endpoint (Supplementary Fig. 11). These data provide evidence of the in vivo activity and efficacy of VIP152 in the treatment of CLL and its potential to improve survival while mitigating disease burden.

## Discussion

Herein we presented the pre-clinical utility of the novel, CDK9-selective inhibitor, VIP152 in CLL using well established assays in primary human CLL samples and a mouse model of this disease. First, we demonstrated that VIP152 is more selective and potent than other inhibitors currently in development. VIP152 had decreased kinase inhibition of CDK7, which was detected with dinaciclib and KB-0742. Recently, it was shown that in a head-on-head comparison VIP152 demonstrated a narrower inhibitory profile for CDK9 than other ‘selective’ CDK9 inhibitors (e.g. Fadraciclib, Alvocidib, KB-0742, and AZD4573) [60]. We observed specific inhibition biochemically and in cells wherein we appreciated a time-dependent decrease in RNA POLII phosphorylation of S2 with no changes in S5 phosphorylation, indicating CDK9-selective inhibition with no measurable inhibition of CDK7 (by absent surrogate POL2 S5 phosphorylation). We demonstrated induction of apoptosis in multiple cell lines, treatment-naïve patient samples, and BTKi/venetoclax relapse/refractory patient samples, and mechanistic evidence that the CDK9 complex is being disrupted. Furthermore, we demonstrate preclinical activity of VIP152 in a novel model of murine CLL. Collectively, these data suggest that VIP152 represents a promising new therapy for drug resistant and up-front treatment of symptomatic CLL.

**Fig. 6:**
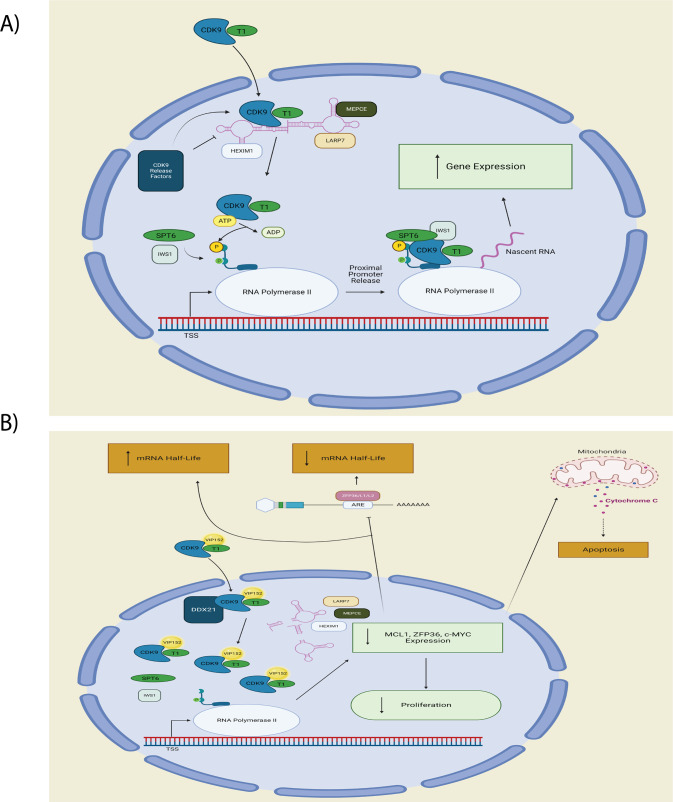
VIP152 mechanistic illustration. **A** Normal signaling pathway for P-TEFb. **B** Proposed signaling pathway for VIP152 inhibition of P-TEFb. VIP152 bound to P-TEFb leads to intranuclear accumulation with dissociation from 7SK RNA complex from DDX21 cleavage. Decreased binding to POLII leads to decreased transcriptional activity with subsequent loss of proliferative signaling, increased apoptosis with loss of MCL1 expression, and increased mRNA half-life from decreased AU-Rich element mediated mRNA degradation subsequent to loss of ZFP36 family expression.

As we have shown, the disruption of P-TEFb’s binding to other protein complexes provides mechanistic justification for the specificity of its activity. Dissociation from the 7SK-RNA complex and nuclear accumulation of P-TEFb were not associated with changes in overall CDK9 levels indicating that VIP152 does not induce P-TEFb degradation but dysfunctional sequestration. Furthermore, the ability of VIP152 to inhibit the CDK9/Cyclin K and CDK9/BRCA1 complexes demonstrates potential mechanisms by which genotoxic stress promotes cytotoxicity [61]. Through loss of POLII association there is a decrease in POLII phosphorylation events, thereby shutting down transcription. Consistent with these findings we appreciated compensatory disruption of ARE-mediated mRNA degradation through downregulation of tristetrapolin and upregulation of ribosomal component transcripts. These findings provide a functional validation of our protein validation data, suggesting a reaction to dismantling the normal transcriptional axis (Fig. 6A, B). The physical decoupling of P-TEFb from POLII may contribute to the downregulation of transcripts seen in our RNA-sequencing. Previously, Zhang et al. showed that CDK9 inhibition could disrupt transcriptional programming without overall downregulation of transcripts; however, their work illustrated an engagement in Lys48 and Glu66 hydrogen bonding through a nitrophenyl substituent [62]. The crystal structure of VIP152 with P-TEFb was recently described and the closest moiety to Lys48 is a para-fluoro substituent, which is proposed to only weakly hydrogen bond with Lys48 [24]. The differences in transcriptional activity may be attributed to overall differences in protein conformational changes induced by different inhibitors and their subsequent effects on protein-protein binding.

The development of CDK9 inhibitors has presented challenges of off-target activity that increase toxicity without therapeutic benefit. Indeed, flavopiridol, being nonselective, and dinaciclib, with moderate selectivity, have shown CDK7 inhibition [21]. As CDK7 regulates the proximal-promoter pause of all POLII-regulated genes, systemic inhibition is an undesirable side-effect [63]. Additionally, the tolerability of these compounds varies, with dinaciclib having shown to be associated with weight loss in vivo [21, 64]. However, VIP152 demonstrates superior on-target and off-target properties with no measured weight loss in treatment groups. The tolerability and highest selectivity of VIP152 suggests that it will have an improved therapeutic index compared with these other inhibitors.

While VIP152 showed activity across a large dose range, human pharmacokinetic data showed a C_max_ of 836 µg/L corresponding to a concentration of 2 µM [28]. These data taken together with previously published clinical trial data of VIP152 suggest it possesses strong clinical potential.

As we have shown, the disruption in binding of CDK9 to RNA Polymerase II machinery after treatment are strong evidence of how VIP152 exerts its effect. Through these data and by also demonstrating the in vivo efficacy and tolerability of VIP152, we maintain that VIP152 represents an attractive therapeutic option for the treatment of CLL. To this point, there is an ongoing clinical trial for the use of VIP152 in patients with relapse/refractory CLL or Richter’s Transformation (NCT04978779).

## Supplementary information

Supplementary Figures & Methods

Supplementary Table 1

Supplementary Table 2

Supplementary Table 3

## Data Availability

All RNAseq and proteomics data have been made publicly available for this manuscript. Additional details may be found in the Supplementary Methods.
