# Supplementary material for: VIP152 is a selective CDK9 inhibitor with pre-clinical in vitro and in vivo efficacy in chronic lymphocytic leukemia
Source: Leukemia. 2022 Nov 14;37(2):326–38. doi: 10.1038/s41375-022-01758-z (PMC9898036; doi:10.1038/s41375-022-01758-z)
Supplement: Supplementary file 1 — Supplementary Figures & Methods [file 41375_2022_1758_MOESM1_ESM.pdf]

Supplementary Figure 1: VIP152 Inhibits GSK3α/β in Biochemical and Cellular Assays. KB-0742, Dinaciclib, Atuveciclib, and VIP152 were tested for their inhibitory capacity against GSK3α/β in the HotSpot Assay (**A-B**) and the NanoBret Assay (**C-D**).

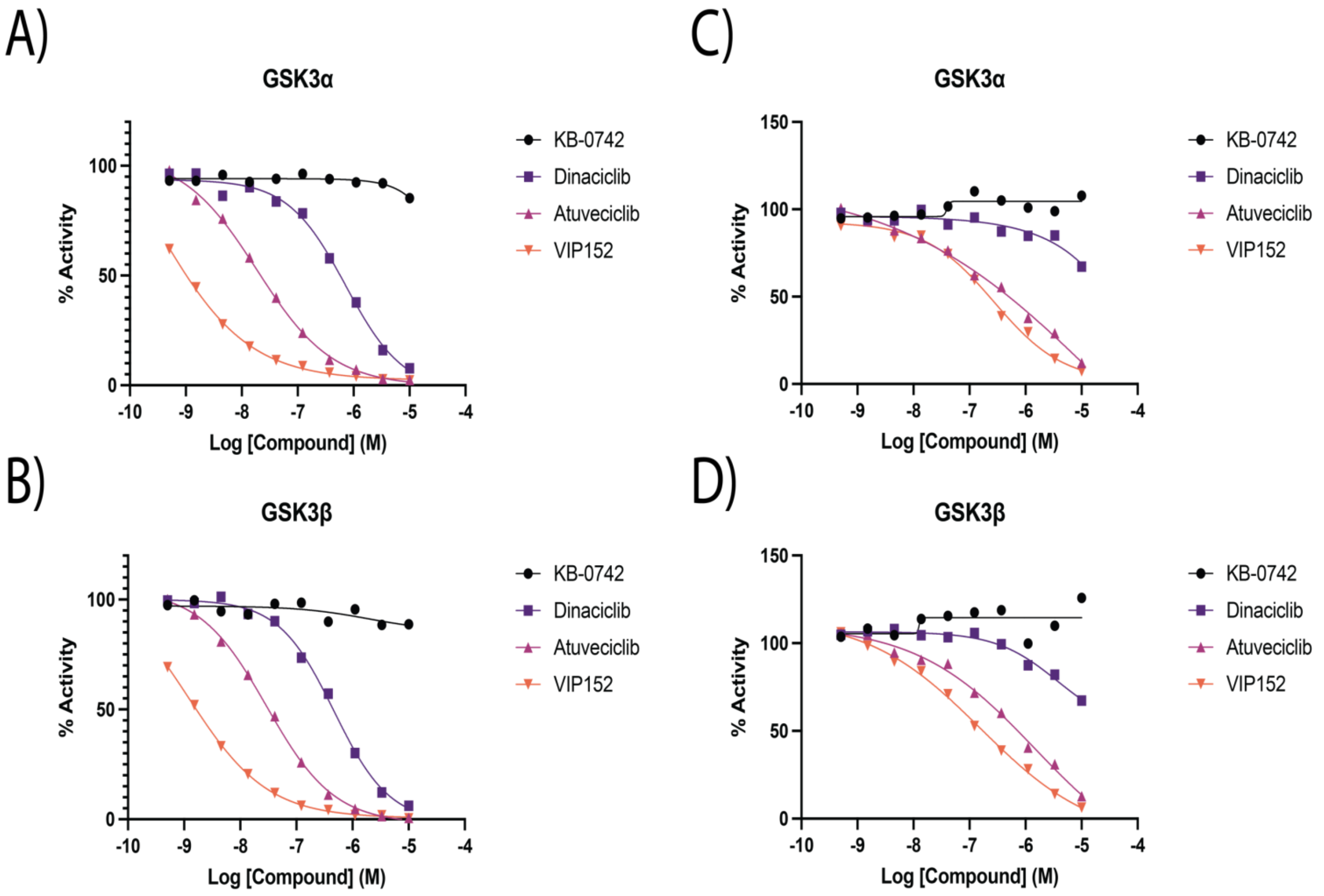

Supplementary Figure 2: VIP152 Decreases RNA POLII Phosphorylation in CLL Cell Lines. HG-3, MEC-1, and OSU-CLL were treated with 1µM VIP152 for 24 hours and protein lysate was obtained at the indicated times.

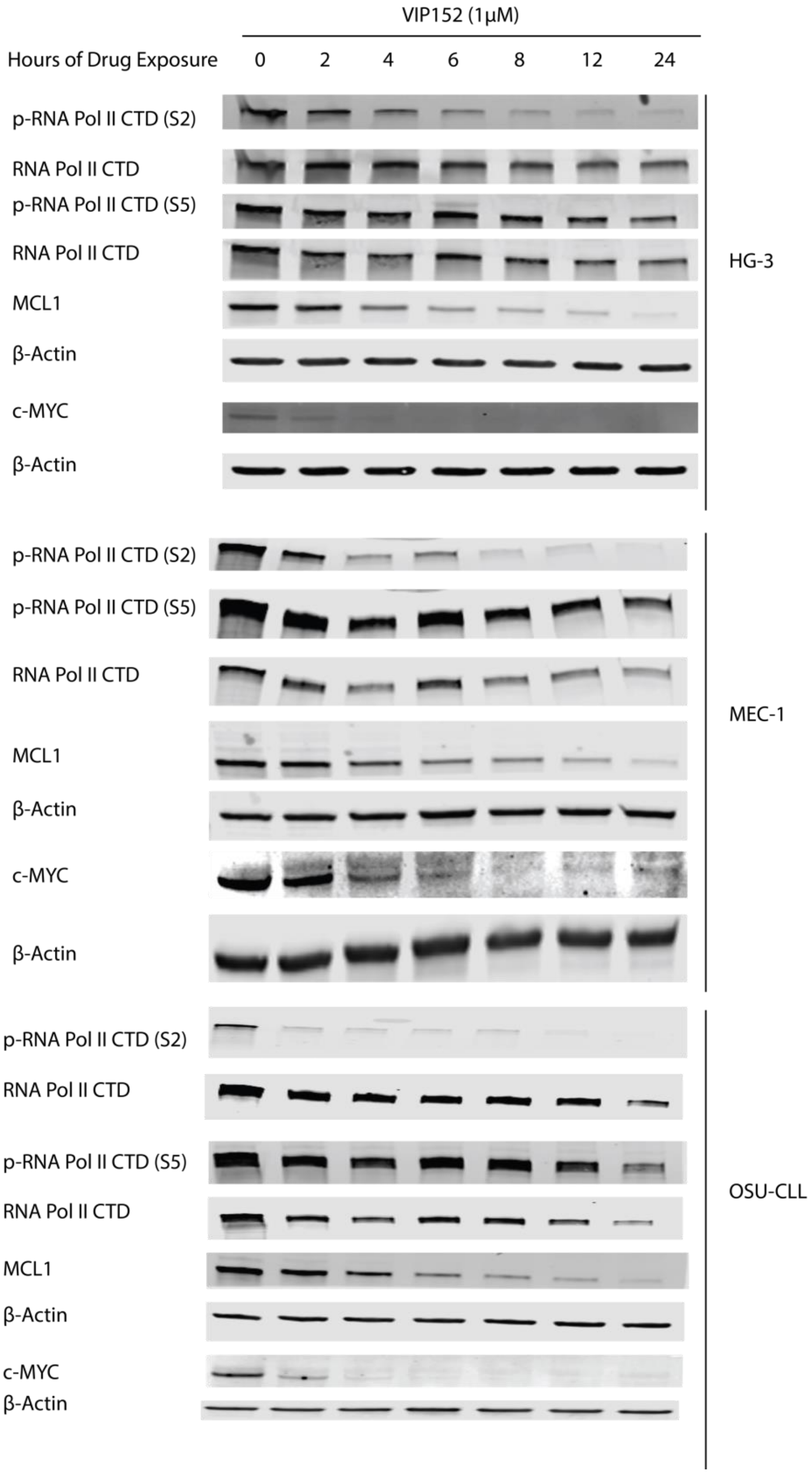

Supplementary Figure 3: Two-Hour Washout Dose-Response Curves. HG-3 and MEC-1 were treated with VIP152 for two hours followed by washout and MTS analysis after 72 hours.

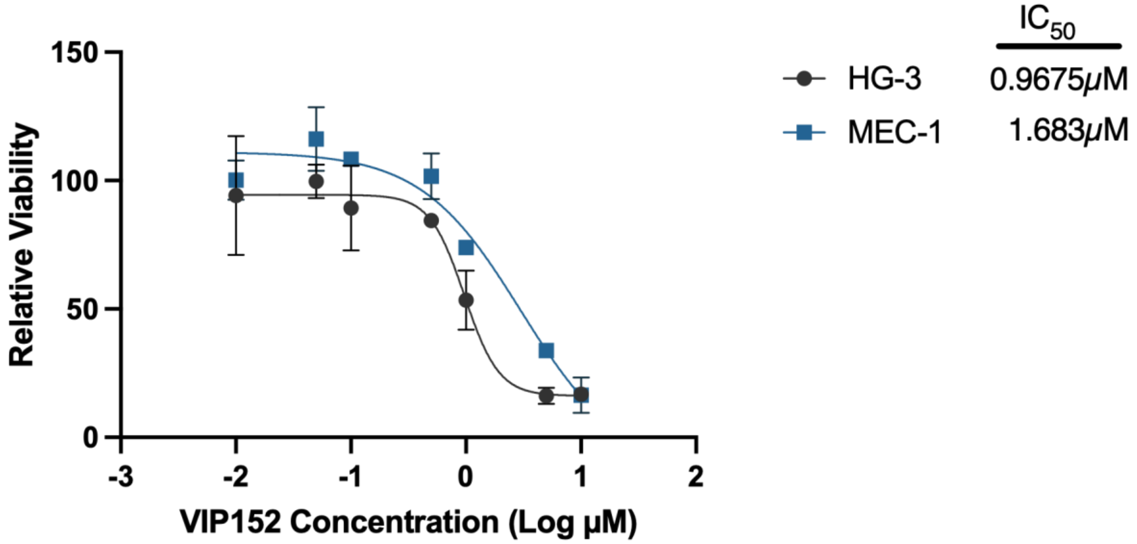

Supplementary Figure 4: CLL Cell Line MTS Analysis. **(A-C)** HG-3, MEC-1, and OSU-CLL cells were plated with VIP152 or DMSO at the concentrations listed for 24hr (**A**), 48hr (**B**), and 72hr (**C**) and analyzed via MTS Assay. **(D-F)** TP53-WT HG-3, R175H-mutant, and R248Q-mutnat cells were plated with VIP152 or DMSO at the concentrations listed for 24hr (**D**), 48hr (**E**), and 72hr (**F**) and analyzed via MTS Assay. Points represent the mean of three biological replicates with three technical replicates per condition and error bars corresponding to SEM.

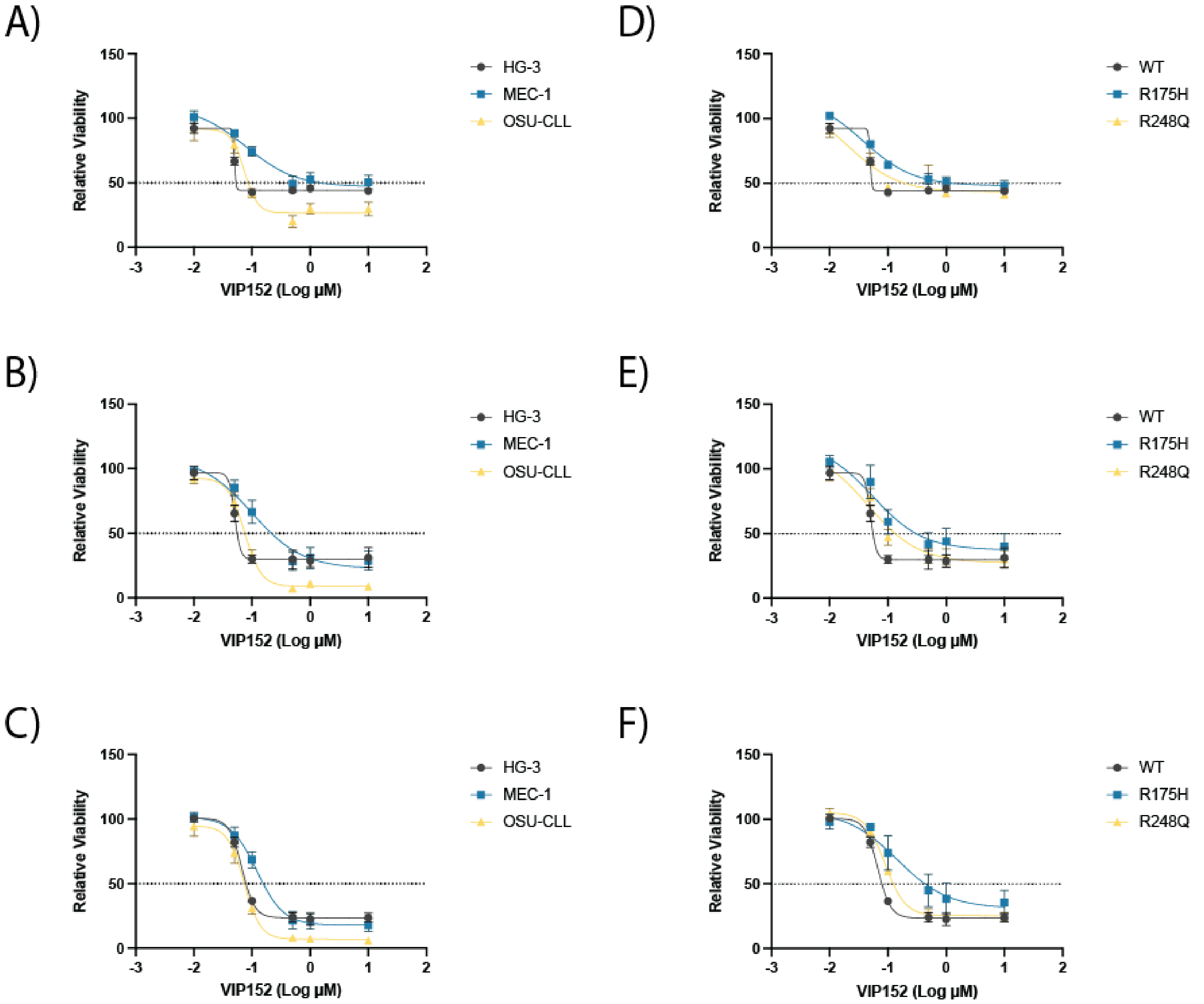

Supplementary Figure 5: Patient Sample Annexin V/PI Flow Cytometry. **(A)** 10 treatment naïve primary CLL samples were treated for four hours with or without stromal support (HS-5) followed by apoptosis analysis. Statistical analysis was performed using a 2way ANOVA with Tukey's multiple comparisons test (*, ** = $p<0.05$ and $p<0.005$ respectively). **(B)** 3 treatment naïve primary CLL samples were treated for four hours without stromal support, then were replated with fresh media and apoptosis was measured after 24 hours. Statistical analysis was performed using a 1way ANOVA with Dunnett's multiple comparisons test (* = $p<0.05$).

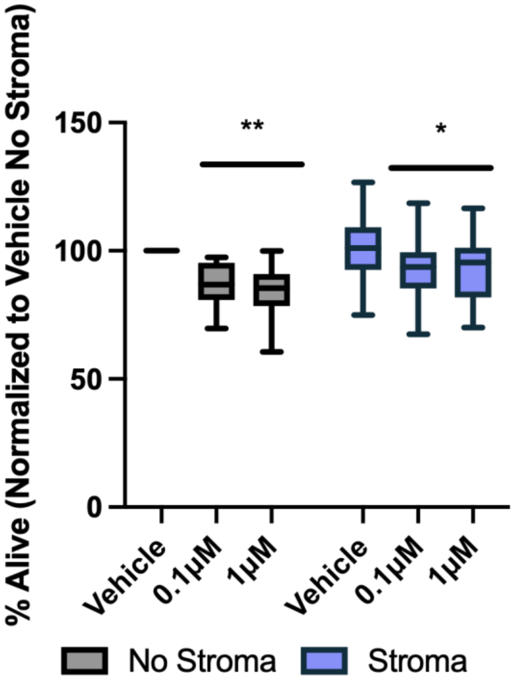

(A)

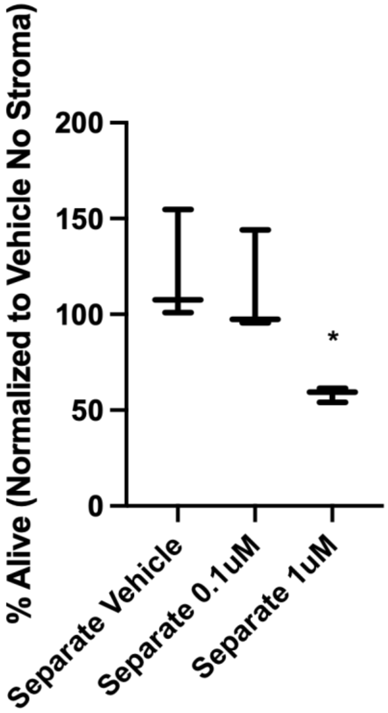

(B)

Supplementary Figure 6: VIP152 Downregulates CDK9 Target Genes and Activates Translation Associated Transcriptional Programming without activating translational machinery. A) Volcano plot of differentially expressed genes comparing VIP152 to DMSO across 4 patient samples. Vertical bars represent a log-fold change of 1 or -1 and the horizontal bar indicates an p-adj. < 0.05. Red dots are genes which are significantly ($p < 0.05$) up or downregulated (LFC >1 or LFC < -1). Blue dots indicate genes which are significantly differentially expressed but do not have a LFC >1 or LFC < -1. Green dots are genes which have a large LFC (LFC >1 or LFC < -1) but do not meet statistical significance thresholds. Gray dots are neither significant nor bear a large LFC. B-D) Gene Set Enrichment Analyses for translation associated transcripts. E) Immunoblot of primary patient samples treated with 1µM VIP152 for 8 hours probing for activation of S6 ribosomal protein. F) qRT-PCR of CLL cell lines treated with 1µM VIP152 for 8 hours (**** = $p < 0.0001$).

A)

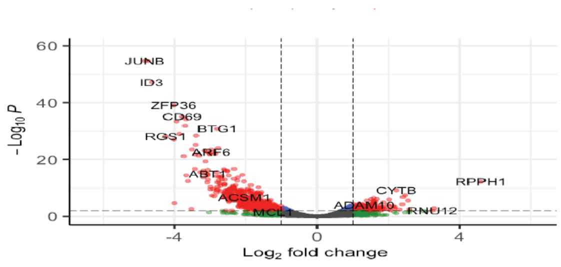

B)

C)

D)

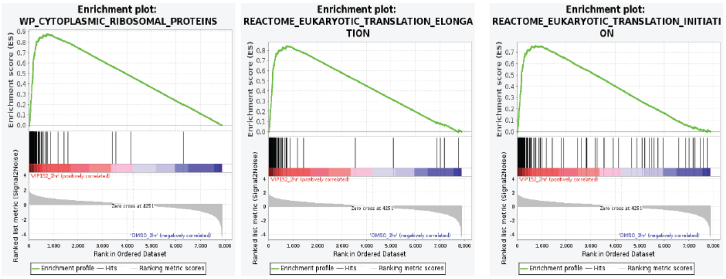

E)

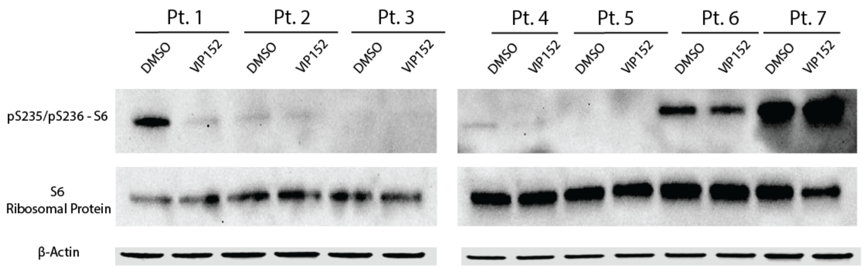

F)

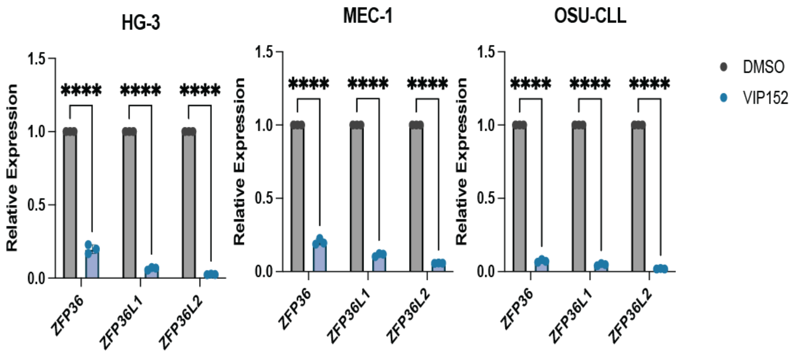

Supplementary Figure 7: CDK9 Inhibition with VIP152 Does Not Prolong mRNA Half-Lives of Two Transcripts Not Bearing AU-Rich Elements. qPCR results of RNA Half-Life studies of GAPDH (**A**) and RPL10A (**B**).

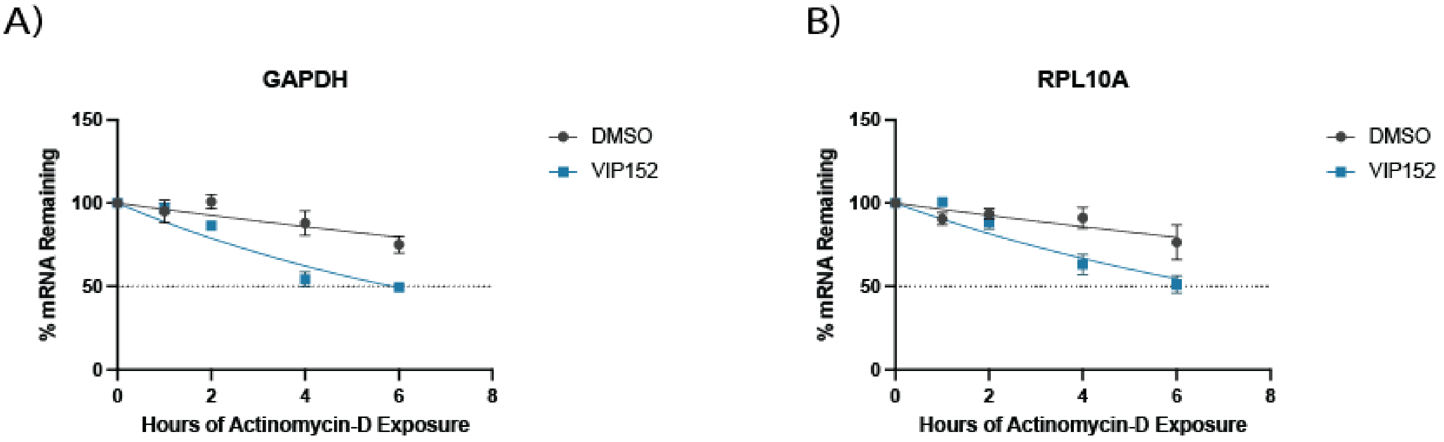

Supplementary Figure 8: Whole Cell Analysis of P-TEFb Expression. HG-3 cells were treated with VIP152 for six hours and lysed with RIPA lysis buffer. Western blot is a representative image of three independent experiments.

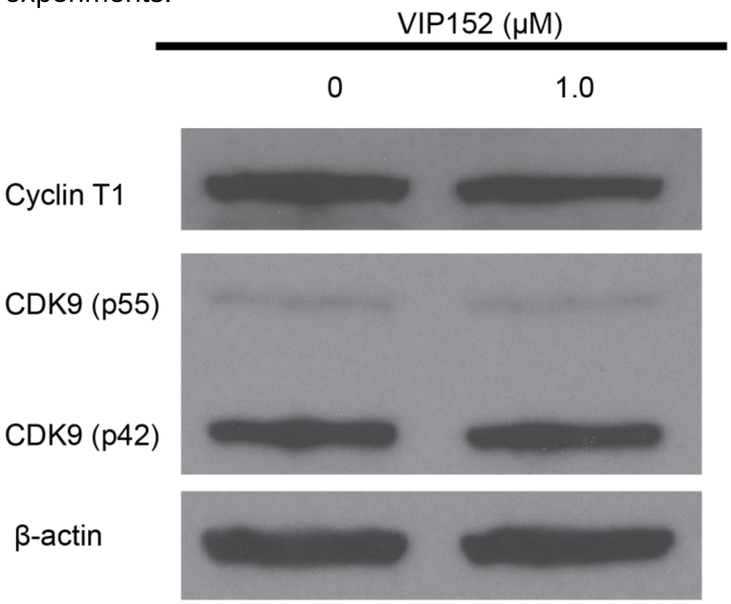

Supplementary Figure 9: VIP152 Treatment Decreases POLII Associated Protein Association. (**A-B**) HG-3 (top) & MEC-1 (bottom) cells were treated for 2 hours with 1µM VIP152. Nuclear lysates were immunoprecipitated with anti-CDK9 antibody and analyzed via Western Blot. (*) indicate long exposure. Western blot is representative of four independent replicates for HG-3 and two independent replicates for MEC-1. (**C-D**) Quantification of POLII western expression normalized to immunoprecipitated nuclear CDK9. Error bars represent SEM.

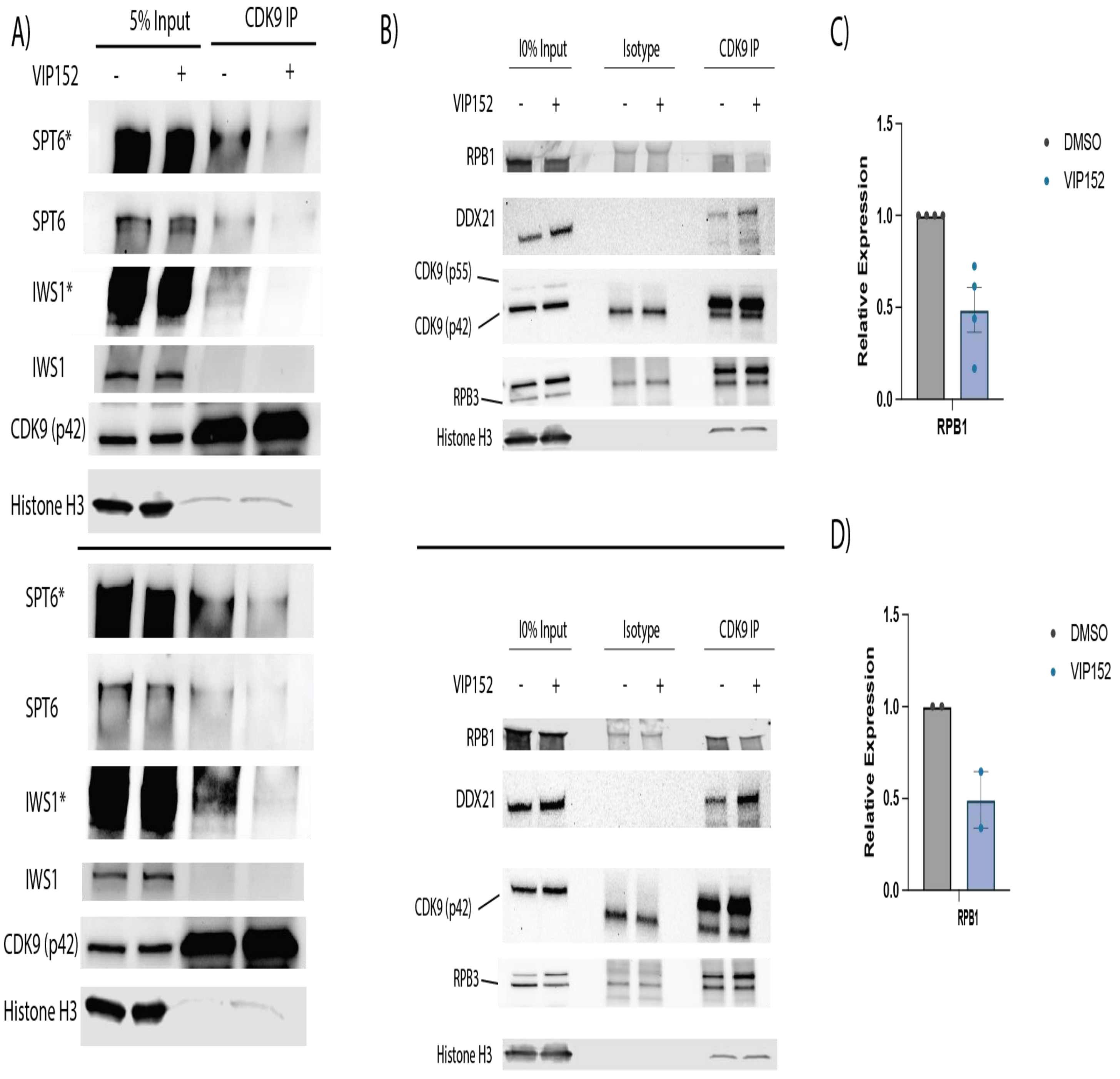

Supplementary Figure 10: VIP152 Treatment Decreases 7SK-RNA Complex Abundance. MEC-1 cells were treated for 2 hours with 1µM VIP152. Nuclear lysates were immunoprecipitated with anti-CDK9 and anti-HEXIM1 antibodies and analyzed via Western Blot.

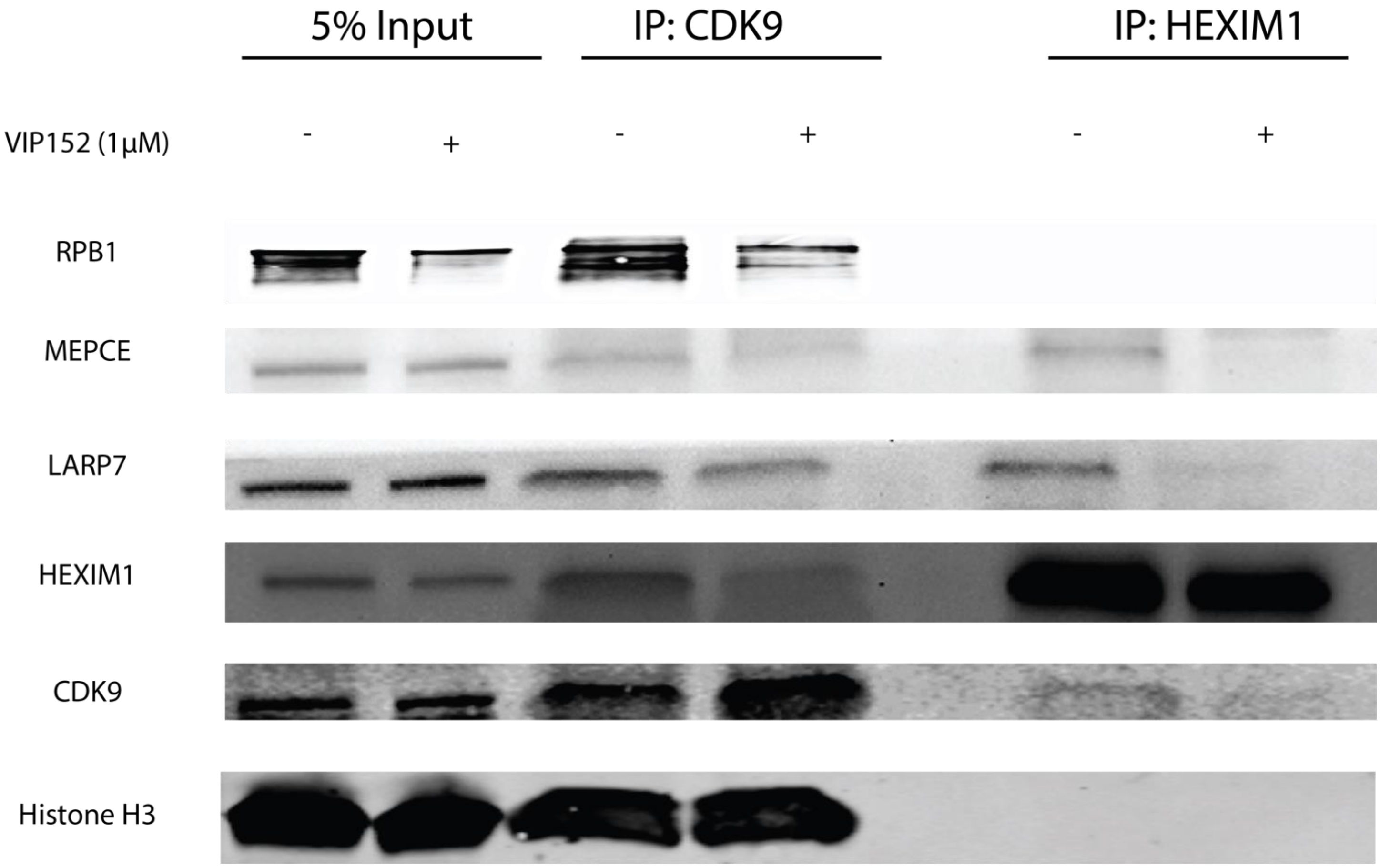

Supplementary Figure 11: Analysis of spleens from Eμ-MTCP1 adoptive transfer study. (A) Spleen weights. **(B)** Spleen homogenate flow cytometry.

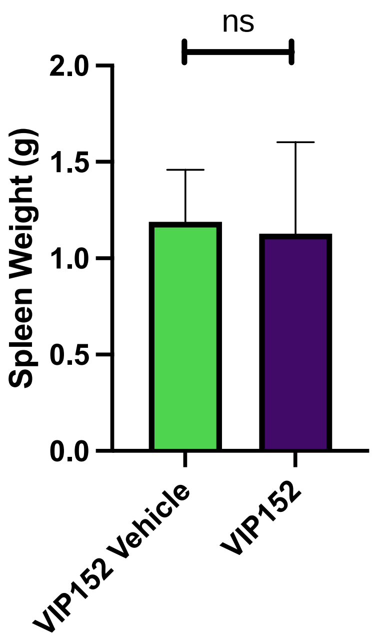

A)

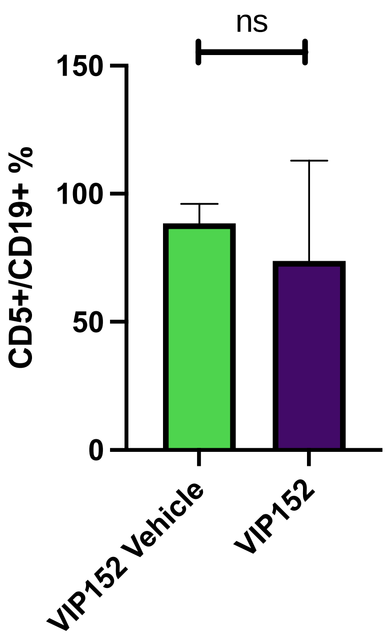

B)

Supplementary Methods

Kinomescan Kinase Profiling Assay

Kinomescan Assay was performed at DiscoverRX and described in Fabian, et al. 2005, Nat. Biotechnol. In brief, kinases were produced and subsequently tagged with DNA for qPCR detection. Streptavidin-coated magnetic beads were bound to biotinylated kinase ligands to serve as a solid support. VIP152 was dissolved in DMSO and tested across a 10 serial threefold dilution set and compared to a test compound and a DMSO control. Kinases which were inhibited by VIP152 would not be bound to the solid-support resulting in decreased substrate for the qPCR reaction. Kinome map was produced via TreeSpot Analysis from DiscoverRX.

HotSpot Kinase Assay Protocol

Compounds tested via HotSpot Kinase Assay were sent to ReactionBio for the experiment. The protocol was described by Anastassiadis T, et al. Comprehensive assay of kinase catalytic activity reveals features of kinase inhibitor selectivity. Nat Biotechnol. 2011 Oct 30;29(11):1039-45. doi: 10.1038/nbt.2017. Kinase substrate was added to reaction buffer (20 mM Hepes (pH 7.5), 10 mM MgCl2, 1 mM EGTA, 0.01% Brij35, 0.02 mg/ml BSA, 0.1 mM Na3VO4, 2 mM DTT, 1% DMSO). Cofactors and kinases were gently added to the reaction buffer. Compounds to be tested were dissolved in 100% DMSO and were delivered into the kinase reaction mixture by Acoustic technology (Echo550; nanoliter range) and incubated for 20 min at room temperature. ^{32}P-ATP was delivered into the reaction mixture and the reaction was allowed to proceed for 2 hours. Kinase activity was detected by P81 filter-binding method.

NanoBRET^{TM} Target Engagement Assay Protocol

Compounds tested via NanoBRET^{TM} Target Engagement Assay Protocol were sent to ReactionBio for the experiment. HEK293 cells were grown to 70-80% confluence appropriately prior to assay and subsequently trypsinized. Lipid:DNA complexes were prepared as follows. A 10µg/mL solution of DNA in Opti-MEM without serum consisting of the following ratios of carrier DNA and DNA encoding NanoLuc fusion were prepared. 9.0µg/mL of Transfection Carrier DNA, 1.0µg/mL of NanoLuc fusion vector DNA, and 1mL of Opti-MEM without phenol red. The solution was mixed thoroughly. Next, 30µL of FuGENE HD Transfection Reagent was added into each mL of DNA mixture to form the lipid:DNA complex and mixed by inversion 10 times followed by a 20 minute incubation at room temperature. Next, in a sterile conical tube, 1 part of lipid:DNA complex was mixed with 20 parts of HEK293 cells in suspension and mixed with gentle inversion 5 times. The cell and lipid:DNA complex was dispensed into a sterile tissue culture dish and incubated for 22-24 hours. Test compounds were then delivered from the compound source plate to the wells of a 384-well white NBS plate by Echo 550. Cells with NanoBRET Tracer Reagent were then prepared by first removing media from dish of transfected HEK293 cells via aspiration, trypsinization, and allowing cells to dissociate from the dish. Trypsin was neutralized using medium containing serum and centrifuged at 200g for 5 minutes to pellet the cells. Cell density was then adjusted to 2E05 cells/mL in Opti-MEM without phenol red. Complete 20X NanoBRET Tracer Reagent was prepared with Tracer Dilution Buffer and dispensed at a ratio of 1:20 Reagent to cells in the tube. The cell

reagent mixture was mixed by inversion 10 times and dispensed into white, 384-well NBS plates and incubated at 37ºC, 5% CO_2 for 1 hour. The plate was removed from the incubator and equilibrated to room temperature for 15 minutes. 3X complete substrate plus inhibitor solution in assay medium was prepared immediately before measuring BRET. 3X complete substrate plus inhibitor solution was added to each well of the 384-well plate and incubated for 2-3 minutes at room temperature. Donor emission wavelength was measured at 460nm and acceptor emission wavelength was measured at 600nm using the Envision 2104 plate reader.

MTS Proliferation Studies, Annexin V/PI Flow Cytometry

Proliferation studies and apoptosis assays were performed as previously described[35]. For the MTS assay using CLL cell lines, 5e5 cells were plated in 50µL of media with 50µL of VIP152-drugged media and cultured using standard conditions. After culturing, 20µL of 5% Phenazine methosulfate (PMS – Sigma Aldrich) in 2mg/mL MTS Reagent (CellTiter 96® AQueous One Solution Cell Proliferation Assay (MTS) – Promega) were added to each well and cells were returned to the incubator for 4 hours. Production of formazan product was measured via absorbance on an HTX-Synergy Plate Reader. For apoptosis assays of primary CLL, cells were acquired under an institutional reviewer board-approved protocol with informed consent. Leukemia cells were isolated from whole blood utilizing RosetteSep Human B Cell Enrichment Cocktail (STEMCELL). Stromal coculture was done by plating HS-5 stromal cells (50% confluent) in a 6-well plate 24 hours before the addition of CLL cells. CLL cells were added to the plate with VIP152 and allowed to incubate for four hours upon which the cells were centrifuged and replated using 50% HS-5 conditioned media. Apoptosis was measured utilizing FITC-conjugated Annexin-V and Propidium Iodide (Leinco) on a Beckmann-Coulter Cytoflex. Primary samples were excluded from analysis if the viability of the DMSO group was <40% as defined by Annexin-V/Propdium Iodide$^{-/-}$ cells. Single-color controls were utilized for compensation using pooled primary cell sample heated for 2 minutes at 50˚C.

Antibodies Used for Western Blotting & Immunoprecipitation Studies

Antibodies used in the presented studies include

- Cell Signaling Technology
    - CDK9 (#2316), Cyclin T1 (#81464), Beta Actin (#3700), RPB1 CTD (#2629), Phospho-Rpb1 CTD (Ser2) (#13499), Phospho-RPB1 CTD (Ser5) (13523), MCL-1 (#94296), c-MYC (#18583), HEXIM1 (#12604), Cleaved PARP (#5625), PARP (#9532), Tristetrapolin (#71632), JunB (#3753), Glycogen Synthase (#3886), Phospho-Glycogen Synthase (Ser641) (#47043), SPT6 (#15616), Histone H3 (#14269), Alpha Tubulin (#3873), Lamin B1 (#13435), IWS1 (#5681), S6 Ribosomal Protein (#2317), Phospho-S6 Ribosomal Protein (Ser235/236) (#4858)
- Abcam
    - RPB3 (ab182150), CDK9 (ab239364), BTG1 (ab151740), DDX21 (ab182156)
- Thermofisher Scientific

- Bethyl Laboratories: LARP7 (#A303-723A), MEPCE (#A304-184A)

qPCR and RNA Half-Life Studies

Patient samples and cell lines were cultured as described above. Samples were drugged for 8 hours with 1µM of VIP152 or DMSO. After 8 hours cells were pelleted at 300 x g for 5 minutes and resuspended in 1mL QIAzol Lysis Reagent (Qiagen). For Actinomycin-D studies, DMSO and VIP152 treatments were supplemented with 500nM Actinomycin-D after 8 hours and samples were harvested as described at 1hr, 2hr, 4hr, and 6hr. RNA was harvested using the RNeasy Mini Kit (Qiagen) according to the manufacturer's recommendations. RNA was measured for purity using a NanoDrop 2000 Spectrophotometer (Thermofisher). cDNA was generated using 2µg of total RNA (or H2O for No-Template Control) with M-MLV Reverse Transcriptase. cDNA was immediately taken for qPCR using TaqMan Fast Advanced Master Mix using Taqman Probes (BCL2 – Hs00608023_m1, ZFP36 – Hs00185658_m1, ZFP36L1 – Hs00245183_m1, ZFP36L2 – Hs00272828_m1, RPL10A – Hs03043870_g1, CELF1 – Hs00198069_m1, 18S (ThermoFisher 4333760F), GAPDH (ThermoFisher 4326317E). Relative gene expression at single-time point (i.e. t_0 DMSO vs VIP152) was calculated using the $2^{-\Delta\Delta Ct}$ method normalizing to 18S rRNA. Half-life calculations were performed by normalizing each housekeeping-normalized treatment to the normalized expression at t_0 and fitting to a one-phase decay using GraphPad Prism. $t_{1/2}$ were calculated using ln(2) / k where k is the decay constant.

Animal Studies

Upon reaching 80% disease, these mice were euthanized and spleens were harvested. Splenocytes from a single donor were then used for the studies described here. 1 x 10^6 splenocytes were again injected via tail vein into male C57BL/6J mice and monitored via flow cytometry as previously described for development of disease as defined by ≥10% $CD45^+/CD5^+/CD19^+$[32]. Upon reaching 10% disease, mice were randomly enrolled into one of two treatment arms: tail vein injection of 12.5mg/kg VIP152 dissolved in (30/10/60 v/v/v PEG400/EtOH/0.5% Tween-80 in water) or vehicle control.

Flow Cytometry analysis of blood

Blood from mice was obtained via cheek bleed according to an approved animal use protocol. 25µL of blood was incubated with the following antibodies obtained from Becton Dickinson & Company (APC Rat Anti-Mouse CD45 – cat. 559864, PE Rat Anti-Mouse CD5 – cat. 553023, BV421 Rat Anti-Mouse CD19, FITC Rat Anti-Mouse CD45R/B220 – cat. 553088). Single color controls and compensation were performed using UltraComp eBeads™ Compensation Beads (ThermoFisher Scientific – cat. 01-2222-42). Blood was lysed eBioscience™ 1X RBC Lysis Buffer (ThermoFisher Scientific – cat. 00-4333-57) and run on a Beckman-Coulter Gallios flow cytometer. Data were analyzed using Kaluza Analysis Software (Beckman-Coulter v2.1).

Database Searching

Mass spectra from all samples (N = 12) were converted to mzML with ProteoWizard and OpenMS (v 2.5.0)[30,31]. Features from converted files were detected with Biosaur and searched on the OpenMS platform with

MSGF+ search engine against a reviewed UniProt human proteome (downloaded 12/07/2020) containing the cRAP and MaxQuant contaminant FASTAs[32]. Search parameters included: full trypsin digest, 1 missed cleavage, carbamidomethylation of cysteine as a fixed modification and oxidation of methionine as a variable modification with precursor and fragment mass tolerances of 10 ppm and 5 Da. PSM rescoring was performed with Percolator and protein inference was performed with Epifany across all samples, setting peptide and protein false discovery rates to 0.05.

Proteomics Data Processing and Differential Expression Analysis

All data analysis was performed with R (version 3.6.2) with various packages and is included in supplemental material (zipped File_S1). Samples were first grouped according to condition and IP-antibody: drug-treated IgG (n = 3), vehicle (DMSO)-treated IgG (n = 3), drug-treated CDK9 (n = 3), vehicle-treated CDK9 (n = 3). and processed for differential expression analysis as described in Gardner *et al*[33]. Briefly, samples were selected for pair-wise comparisons prior to filtering out lowly expressed proteins and contaminants (lowly expressed proteins were defined as having fewer than 3 total observations or total abundance < 2^15 (32768)), and missing values were imputed with a multiple imputation approach by treatment group. Data was quantile normalized, and significance (*p*-value < 0.05) determined by a modified exact test. Downstream gene ontology (GO), KEGG and pathway analyses utilized the list of significant proteins identified. For GO and KEGG, proteins were filtered by direction of the log fold-change to produce protein lists specific to that condition. Mass spectrometry data is available through the mass spectrometry interactive virtual environment (massIVe) at (ftp://massive.ucsd.edu/MSV000089137/).

Data Accession

All data associated with these experiments have been made publicly available.

RNAsequencing data has been deposited to GEO (GSE199037; reviewer code: ahexmesinjijxyn)
https://www.ncbi.nlm.nih.gov/geo/query/acc.cgi?acc=GSE199037

For proteomic data: The URL for the dataset is: ftp://MSV000089137@massive.ucsd.edu Reviewer instructions can be found here:
https://ccms-ucsd.github.io/MassIVEDocumentation/#reviewer_access/
Username: MSV000089137_reviewer
Pwd: 4_r3v13w3r$
